# Neuropsychiatric and Behavioral Involvement in AAS Abusers. A Literature Review

**DOI:** 10.3390/medicina55070396

**Published:** 2019-07-22

**Authors:** Giuseppe Bertozzi, Monica Salerno, Cristoforo Pomara, Francesco Sessa

**Affiliations:** 1 Department of Clinical and Experimental Medicine, University of Foggia, 71122 Foggia, Italy; 2 Department of Medical, Surgical and Advanced Technologies “G.F. Ingrassia”, University of Catania, 95121 Catania, Italy

**Keywords:** anabolic androgenic steroids, side effects, lifestyle, neuropsychiatric manifestations, behavior

## Abstract

*Background and Objectives:* Anabolic androgenic steroids (AASs) are a complex group of molecules that include both steroidal androgens and synthetic compounds, derived from testosterone. AASs are commonly used to support pharmacological therapy in cases of primary or secondary hypogonadism, major burns, and neoplastic cachexia. Their prolonged and supra-physiological consumption can provoke several adverse effects on various organs and systems. Among these, the physiopathological mechanisms that induce neuropsychiatric disorders related to AAS abuse are poorly known. For this reason, the proposed review aims to retrace the pathway of action of testosterone to focus on the effects on the central nervous system and specifically highlight the effects of AASs on neuropsychiatric and behavioral functions, as well as on lifestyle. *Materials and Methods:* This review was conducted using PubMed and Google Scholar databases. On these database websites, we searched for articles from 1 January 1980 to March 2019 using the key terms: “AAS,” “Anabolic Androgenic Steroids,” “brain,” and “neurology.” *Results:* The use of AASs through self-administration yields circulating androgens levels, inducing neuron apoptosis, which is linked to thinner cortex and, in general, less cortical volume. The same alterations affect the putamen. These differences were more evident when correlated with longer use. From a functional point of view, prolonged AAS consumption seemed to be related to lower connectivity between amygdala and frontal, striatal, limbic, hippocampal and visual cortical areas. On the other hand, AAS use seems to negatively condition the positive effects of the sport exercise, reducing its important anti-apoptotic and pro-proliferative functions on the hippocampus, implicated in anxiolytic control. *Conclusion:* This review clarifies the major aspects of the side effects related to AAS use/abuse highlighting the complex mechanisms on neuropsychiatric and cognitive pathological alterations and also the emotional and behavioral dysfunctions.

## 1. Introduction

AASs (anabolic androgenic steroids), largely known as anabolic steroids, are a complex group of molecules that include both steroidal androgens, such as testosterone and its related-precursors (dehydroepiandrosterone, androstenedione, and androstenediol), as well as many different synthetic compounds. Physiologically produced by Leydig cells and the adrenal cortex in men, and by preovulatory follicles or the corpus luteum and adrenal cortex in women, testosterone is the major anabolic androgen in the human body.

Therefore, long-term use is associated with a wide range of pathological disorders, which affect the liver, as well as cardiovascular, reproductive, musculoskeletal, endocrine, renal, immunologic, and neuropsychiatric functions [1,2,3]. Amid these, neuropsychiatric and behavioral disorders related to AAS abuse are poorly known. In this context, AAS users experience biphasic sensations; on the one hand positive mood, more energy and better self-confidence, while on the other hand depression, irritability, anxiety, fatigue, and insomnia, above all during withdrawal periods [4].

For this reason, the proposed revision aims to retrace the pathway of action of testosterone to focus on the effects on the central nervous system and specifically highlight, through a collection of studies conducted on humans, the effects of AASs on neuropsychiatric and behavioral functions, as well as on the lifestyle. In fact, AASs influence the organization and development of brain circuits as well as the neuroendocrine system functioning.

## 2. Materials and Methods

### 2.1. Research Strategy

This review was conducted using PubMed and Google Scholar databases. On these database websites, we searched for articles from 1 January 1980 to March 2019 using the key terms: “AAS,” “Anabolic Androgenic Steroids,” “brain,” and “neurology.”

### 2.2. Study Selection

Articles were included in the review according to the following inclusion criteria: publication in peer-reviewed journals and published between 1 January 1980 to March 2019. Case reports were excluded from the analysis.

**Figure 1:**
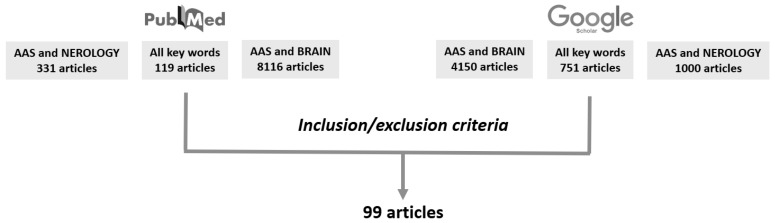
The research strategy used for the literature review.

Articles were excluded by title, abstract, or full text for irrelevance to the investigated issues: AASs, neurological structures, and AAS and lifestyle. Lastly, to identify further studies that met the inclusion criteria, the references of the selected articles were also reviewed (Figure 1).

## 3. Results

### 3.1. Pathophysiology of Testosterone and AASs

Testosterone has several functions; in particular, because of its effects on the body, it influences sexual differentiation, its secretion being greater in men than in women. The biochemical pathway to produce testosterone starts from cellular cholesterol, which after an enzymatic cleavage of the lateral chain becomes pregnenolone. This latter molecule can follow two different metabolic patterns. In the first 3-beta-hydroxysteroid dehydrogenase transforms pregnenolone into progesterone, then 17-a-hydroxylase adds an oxidrile group to pregnenolone and progesterone, synthesizing, respectively, 17-a-OH-pregnenolone and 17-a-OH-progesterone. Acting on each of these molecules, 17,20-lyase, produces dehydroepiandrosterone (DHEA) and androstenedione. Androstenediol and testosterone are produced by the action of 17-beta-hydroxysteroid dehydrogenase on the two molecules. Androstenediol, moreover, can be transformed into testosterone by 3-beta-hydroxysteroid dehydrogenase [5,6,7].

Testosterone can exert its action itself but, more commonly, via the action of two enzymes called 5-a-reductase and CYP19-aromatase; in fact, it is possible to distinguish active (di-hydro-testosterone and estradiol respectively) and inactive (androsterone and etiocholanolone respectively) testosterone metabolites. In particular, di-hydro-testosterone and testosterone, acting by means of the binding androgen receptor, have a different affinity, the first being higher than the second. As has been stated above, testosterone is the main androgen in men, primarily produced by Leydig cells and, in less quantity, by adrenal glands, in a relatively constant amount, with some diurnal variation that is age correlated. Indeed, diurnal variations of testosterone are well documented with specific levels that peak between 05:30 and 08:00 and trough levels occurring approximately 12 h later [7,8,9,10,11]. Some studies have demonstrated that the increase in saturation of binding proteins is followed by a rise in testosterone production. This modification and the small but significant protein concentration changes are the most important determinants in modifying rhythm amplitude. In addition, it has been supposed that the increase in cortisol concentration can explain the early-morning decline in the non-sex hormone-binding globulin (SHBG)-bound fraction [10]. Androgens have several actions in the human body, in particular, in males, they deal with body growth and sexual differentiation acting on all tissues and organs. Testosterone production, after a role in fetal development, starts its effect during puberty, under the metabolic stimuli of gonadotropins, regulating, above all, spermatogenesis [12,13].

In fact, the extra-hypothalamic central nervous system, collecting physiologic and psychologic stimuli from the internal and external world, regulates, in different ways, the activation of the hypothalamus, causing a pulsatile release of gonadotropin releasing hormone (GnRH) into the portal system. This system links the hypothalamus to the pituitary glands, determining a specific action on the anterior part, stimulating the release and new synthesis of luteinizing hormone (LH) and follicle stimulating hormone (FSH), which, in turn, act on Sertoli cells, controlling testosterone secretion and promoting spermatogenesis [14,15,16]. From the chemical point of view, testosterone is a steroid hormone, thus it cannot flow totally free in the blood; indeed, about 98% can be found in the blood bound either to SHBG or albumin. The remaining unbound 2% is free to act, passing through the cell membrane, regulating and deterring the related metabolic effects. In this sense, SHBG and albumin changes have an important repercussion on testosterone action, influencing its circulating amount [17]. In fact, binding to SHBG protects steroid hormones from metabolic degradation. On the other hand, a small fraction of free androgen-like hormones is present in the plasma, which can passively pass through the hematologic barrier due to their lipophilic nature. In the encephalic field, however, there is also a significant contribution of de novo synthesized hormones from the parenchyma, such as 17β-estradiol, testosterone, or dehydroepiandrosterone, acting with intracrine, paracrine, and autocrine functions [18]. High levels of circulating androgen results in a reduction in the release of gonadotropins, T3, and T4, as well as thyroid binding globulin, and an augmentation of thyroid stimulating hormone [19].

As has been reported above, there are three main pathways through which testosterone can act: (i) Directly on the androgen receptor, (ii) via dihydrotestosterone (DHT) produced by the action of the 5-a-reductase, and (iii) via the estrogen receptor by means of estradiol produced by CYP19 aromatase [8]. A recent literature review conducted by Moraga-Amaro et al. reported several studies demonstrating the presence of specific receptors for sexual steroid hormones (AR and ER) expressed in the cerebral parenchyma using PET [20].

It may be expected that the use of AASs through self-administration combined with physiological production, both by men and women, yields circulating androgens levels, which are orders of magnitude higher than the normal physiological levels. AAS effects are strictly linked to the age of the abuser. Several studies in humans suggest that adolescents may be more sensitive than adults [21] and females may be more sensitive than males [22].

The main effect described in human abusers is significant and often permanent changes in sexual behaviors and reproductive state have been described [3]. The adverse effects of AAS use/abuse on the reproductive system are suppression of gonadal steroidogenesis, amenorrhoea, clitoral hypertrophy, testicular atrophy, disproportionate growth of the inner prostate, and masculinization of female fetuses [23,24].

Other organs that are involved in the adverse effects after AAS assumption are the liver and cardiovascular systems. Several studies have described damage to liver tissue, such as impaired function, hepatic cholestasis (bile canal obstruction) causing jaundice, peliosis hepatitis (blood-filled sacs in the liver), and liver tumors (increased risk) [25,26].

Moreover, the cardiovascular system can be considered as one of the most important systems involved in the evaluation of AAS abuse damage. Several diseases are well described in AAS abusers: Increased risk of thrombotic events such as myocardial infarction or stroke (raised LDL, lowered HDL and apolipoprotein-1, raised hematocrit (due to polycythemia), and lowered plasma fibrinogen) cardiac damage (left ventricular hypertrophy, fibrosis, and heart failure), and sudden cardiac death [3,25,26,27,28,29].

Finally, it is very interesting to analyze the main adverse effects of AAS use/abuse and the central nervous system. Indeed, increased libido in men and women, which may be difficult to control, hypomania (a less severe form of mania), heightened irritability, increased aggression and hostility, destructive impulses, self-destructive impulses, and severe depression [21,25,26].

### 3.2. Neuropsychiatric Involvement of AAS

First used by elite athletes, AAS use has developed within the general population not only for ergogenic purposes to improve performance but also for cosmetic use to enhance personal appearance [25,26,27,28,29]. Perhaps following new trends in the idealization of the male body and reflecting the prevalence of “muscle dysmorphic disorder” that has become so widespread to be included in DSM-V, AAS consumption seems no longer a prerogative of athletes or weightlifters [30,31,32,33]. In fact, this body misperception was identified as an independent risk factor to start AAS use, furthermore, this use is not reduced even after the increase in muscle mass [34]. However, this relationship, between AAS use and either psychiatric or behavioral disorders, seems to be always more biunivocal. In fact, Kanayama et al. demonstrated among adolescents that a diagnosis of psychiatric disorders, such as conduct disorder and sociopathy, is the substrate of AAS use and promotes the switch from use to abuse [34]. On the other hand, AAS use can lead to profound effects on the brain and behavior. Poor impulse control with aggressive behavior, anxiety, extreme mood swings from depression to mania or hypomania have been linked to AAS use over the past few decades [35,36,37,38,39].

Bitran et al. in 1993, demonstrated that testosterone propionate administered to adult male mice in a moderate concentration for only a 6-day period relieved an increase in anxiety-like behavior [40]. Minkin et al. showed similar results with nandrolone decanoate during an 8-week period of administration, recently confirmed in a study by Ambar and Chiavegatto about nandrolone decanoate administration in rats and mice [41,42]. An analogous description can be made according to AAS abuse and aggressive behavior in male rodents, with degree changes in relation to the chemical nature of the substance [43,44].

**Figure 2:**
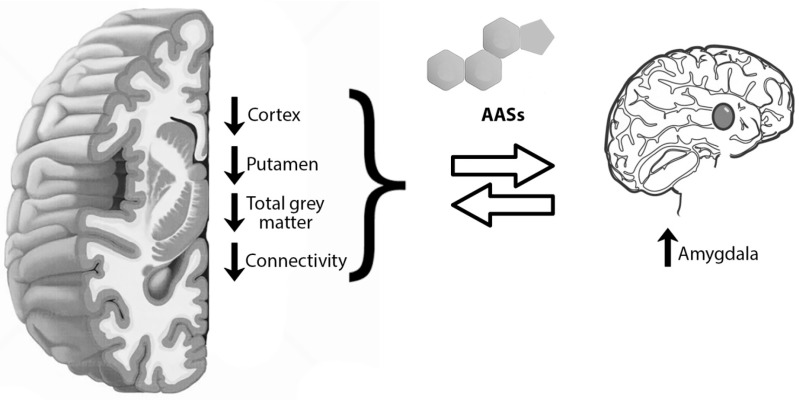
Anabolic androgenic steroids (AASs) vs. brain: AASs have been associated with anatomical and functional brain alterations. From the anatomical point of view, AAS-induced neuron apoptosis is linked to thinner cortex and less cortical volume. The same alterations affect the putamen, associated with less total grey matter. These differences were more evident when correlated with longer use. Frontal, parietal, temporal, and occipital cortex were thinner in long-term consumers than in short-term ones. On the contrary, the right amygdala was enlarged. From a functional point of view, prolonged AAS consumption seemed to be related to lower connectivity between amygdala and frontal, striatal, limbic, hippocampal and visual cortical areas, also involving the DMN (default-mode network), and a complex composed of the superior and inferior frontal gyri (SFG/IFG) and the anterior cingulate cortex (ACC).

This substance, indeed, has the characteristic to pass the blood-brain barrier freeing its actions directly on the nervous system inducing brain alteration [45]. Testosterone and AAS can act at this level via AR largely distributed in the cerebral cortex, hypothalamus, hippocampus, brain stem, and amygdala [46,47,48,49]. These nervous structures play an important role in the regulation of higher functions, such as emotion and cognition. Moreover, these molecules can act even with a non-genomic mechanism influencing GABAergic, glutamatergic, or serotoninergic patterns. Thus, supraphysiological testosterone and AAS doses can be associated with apoptosis of a wide spectrum of mammalian cells, including neurons, maybe through amyloid storage or increased oxidative stress (Figure 2) [50,51,52]. Bjørnebekk et al. conducted a study on 150 participants divided into 82 current or previous AAS users and 68 non-users having undergone the Magnetic resonance imaging (MRI), and demonstrated that AAS users had a thinner cortex and in general less cortical volume than non-users [53]. These differences were more evident when correlated with longer use. Frontal, parietal, temporal, and occipital cortexes were thinner in long-term consumers than short-term ones. Analogous alterations were seen at the expense of the putamen, one of the structures of the basal ganglia, which can be associated with less total grey matter rather than a specific functional alteration in the area. On the contrary, a previous study on brain morphometry linked AAS use to an enlargement of the right amygdala [45] and of the nucleus accumbens [54]. Thinner cortex areas and augmentation in nucleus accumbens dimensions were proven even in cannabinoid and alcohol abuse [55,56], maybe explaining the anatomical substrate for the development of drug addictive behavior.

Furthermore, a small-group study on the brain network, from a functional point of view, using for this purpose functional MRI proved the lower connectivity between the amygdala and frontal, striatal, limbic, hippocampal, and visual cortical areas in prolonged AAS consumption and abuse [45]. Heany et al. succeeded in demonstrating activation foci in the amygdala after higher testosterone concentrations, either endogenous or exogenous [57]. Based on these evaluations, Westlye et al. performed fRMI on 151 subjects, 82 current or previous AAS consumers and 69 non-users, documented alterations in connectivity between the amygdala and the default-mode network (DMN) [58], which has been connected to various psychiatric and neurological conditions [59]. In addition, lower connections were revealed between the dorsal attention network (DAN) and the system composed of the superior and inferior frontal gyri (SFG/IFG) and the anterior cingulate cortex (ACC). DAN is an attention core composed, in turn, of structures located in the intraparietal sulcus (IPS), superior parietal lobule (SPL), and the frontal eye field [60]. SFG is a structure assigned to executive functions, such as working memory and cognitive control [61]. Right IFG, instead, has the role of impulse suppressor and controller [62,63]. ACC, lastly, does not conduct a concrete function but contributes to the cognitive, motor, and attentional control or to reward-based decisions [64,65]. Abnormalities in this region were also demonstrated with magnetic resonance spectroscopy (MRS) as a response to glutamate-turnover increase [45].

Seitz et al. in order to assess effects derived from AAS use on white matter, subjected 9 long-term AAS users and 8 non-user controls to diffusion tensor imaging (DTI), measuring the fractional anisotropy (FA) as an index of the white-matter organization [66]. In this way, they focused their study on inferior-front-occipital fasciculus (IFOF), key bundles in the amygdala network, involving the orbitofrontal and ventromedial frontal cortex, the middle and inferior frontal gyri, and the ventral temporal and occipital regions, with implications in executive functions, in circuitry related to the ability of reading and language and to cognitive control and/or drug dependence [61,67,68,69,70]. Microstructural abnormalities were detected as increased FA in AAS users versus AAS non-users, and these signals were even greater in relation to the dose [66].

Reduction in orbitofrontal cortex (OFC) activity can explain the lack in inhibitory control, resulting in aggression and violent behavior, drug addiction and obsessive-compulsive disorder as a consequence of androgen consumption [71].

### 3.3. AAS Use vs. Exercise and Diet

**Figure 3:**
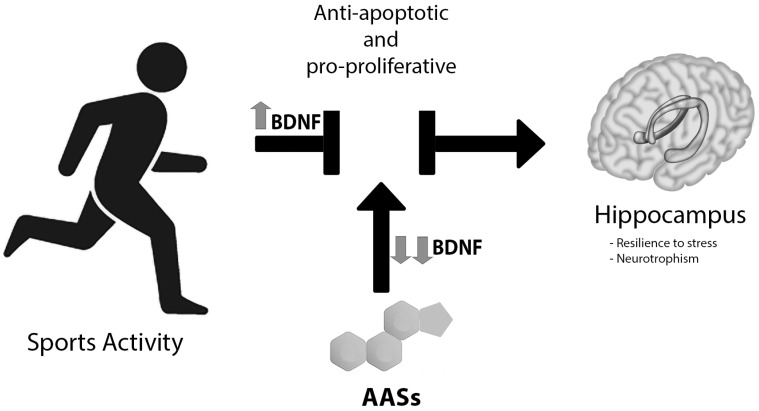
AASs vs. sport exercise: The latter determines important anti-apoptotic and pro-proliferative functions on the hippocampus, implicated in anxiolytic control. AAS consumption blocked this effect, likely via brain-derived neurotrophic factor (BDNF), mRNA expression is inversely dependent on AAS use. Moreover, exercise seems to not be capable of repairing hippocampal AAS-induced damage. The result is a significant lowering of the hippocampal levels of BDNF, reducing adaptability to stress and neurotrophism.

Testosterone and ASS have been confirmed to be increased in anxiety-like behavior when administered to adult male mice even in moderate concentrations for short periods. On the contrary, previous reports showed that rodent voluntary wheel running decreases anxiety-like behaviors, while exercise, alone, has been demonstrated to decrease anxiety in humans [72,73,74]. According to Novaes Gomes et al., exercise acts as an important anti-apoptotic and pro-proliferative factor on the hippocampus that was implicated in anxiolytic control, an effect that was compromised by AAS consumption in adult male rats (Figure 3) [75]. A key molecule to elicit this result is brain-derived neurotrophic (BDNF), whose mRNA expression is inversely dependent on wheel running and AAS use [76]. Moreover, exercise seems to increase BDNF mRNA synthesis and BDNF protein production in the hippocampus, with consequential improved behavior [76]. On the contrary, Tanehkar et al. proved that male rats subjected to exercise presented higher levels of BDNF-expression, however, this was not able to repair the hippocampal AAS-induced damage [77]. On the contrary, high doses of AAS are associated with lower concentrations of BDNF in the hippocampus and prefrontal cortex, which can be linked with a reduction in glucocorticoid receptor expression [78,79]. Therefore, the result is that the sum of the intake of large doses of exogenous steroids and immobilization leads to a significant lowering of the hippocampal levels of BDNF, reducing adaptability to stress and neurotrophism.

Moreover, testosterone and AAS, likely via ROS-production, play an important role in multi-organ damage, above all with cardiac and neuropsychiatric consequences. On the contrary, it is known that short-term diet and moderate exercise can modulate ROS production, antagonizing this AAS-derangement aspect, which is responsible for the increase of cardio-vascular risk and brain dysfunctions [80].

In association with exercise to increase skeletal muscle mass, AAS consumption is often associated with a high protein diet integrated by supplements such as casein and whey powders, and with the aim to minimize the natural catabolic processes generated by strenuous exercise [81,82]. High protein diets are related to complex agonistic interaction between peptide YY3-36 (PYY3-36) and NPY Y2 receptors; receptors that are co-expressed in the amygdala and responsible for causing anxiety, in addition, to modulating signaling belonging to the mesolimbic dopaminergic reward pathways [83,84].

Furthermore, to these dietary restrictions, the ingestion of “fat supplements” has to be added, in order to induce weight loss and improve performance, via the modification of lipid parameters, the promotion of fat metabolism, the augmentation of testosterone synthesis, and the storage of glycogen [85]. Among these supplements conjugated linoleic acid (CLA) is the most studied, with the ability to increase strenuous exercise measured as the distance run by mice until the onset of muscle fatigue. Thus, this improvement was not because of mitochondrial-DNA synthesis, rather by means of inducing type-IIx fiber hypertrophy in the plantaris muscle [86].

## 4. Discussion

Nowadays, the market for doping drugs is huge and constantly increasing. The phenomenon of doping no longer affects only professional athletes, but also subjects who perform amateur sports activities. Current legal restrictions are not enough to stop this continuously expanding phenomenon. This illegal practice, not justified by pathological conditions, can provoke several adverse effects, most frequently on the cardiovascular system. This review clarifies the major aspects of the side effects related to AAS use/abuse highlighting the complex mechanisms on neuropsychiatric and cognitive pathological alterations, and also the emotional and behavioral dysfunctions. As far as these aspects are concerned, they are very far from finding a complete explanation through an anatomo-functional organic alteration or a clear chemical–molecular pathway.

To date, it is clear that AAS use/abuse provokes greatly debilitating manifestations, such as poor impulse control with aggressive behavior, anxiety, extreme mood swings from depression to mania or hypomania; even the detoxification of AAS abusers represents a poorly studied area [87]. In this regard, a recent review conducted by Medras et al. stressed that during the detoxification phase of AAS abusers somatic and psychic disorders may develop, related to the sudden reduction of circulating testosterone levels [88]. Among these, depression, with suicidal ideation, and sexual dysfunction due to hypogonadism, libido recovery, and erectile dysfunction, are certainly the most serious [85,86,87,88]. As far as behavioral disorders are concerned, these can be related to organic alterations that are able to determine neuroadaptive changes whose result is a compromised ability to manage stressful conditions. These elements, in turn, expose users to the risk of developing a substance abuse disease. In this regard, it can be very useful to analyze the relationship between genetic predisposition for stress management and AAS use/abuse [89,90]. In other words, further studies are needed to highlight the possible relationship between the subject with a negative genetic predisposition to stress management and AAS abuser.

## 5. Conclusions

In conclusion, physicians should begin to be aware of the dimensions of AAS abuse and the diagnostic pitfalls but especially the therapeutic difficulties. In fact, people who abuse AAS appear to be vulnerable, presenting more complex internal and interpersonal dynamics than not dependent subjects [23,24,91,92]. However, if it is true that the dependence on AAS shares some lifestyle characteristics with other illicit substances, both neurobiological and behavioral alterations [93,94], the underlying mechanisms remain unknown. As with other addictions, there will be a combination of premorbid conditions and uncontrolled consequent use. Some studies have shown that testosterone administration has the power to alter the emotional sphere, in particular concerning fear, anger, and disgust [95,96,97,98,99]. These alterations can explain neuropsychiatric and behavioral personality modifications. Further studies that will shed light on these complex connections, especially in the clinical setting, are necessary.
